# Isolation and Identification of Porcine Deltacoronavirus and Alteration of Immunoglobulin Transport Receptors in the Intestinal Mucosa of PDCoV-Infected Piglets

**DOI:** 10.3390/v12010079

**Published:** 2020-01-09

**Authors:** Shaoju Qian, Xiangchao Jia, Zitong Gao, Weida Zhang, Qingrong Xu, Zili Li

**Affiliations:** 1 State Key Laboratory of Agricultural Microbiology, College of Veterinary Medicine, Huazhong Agricultural University, Wuhan 430070, China; qianshaoju@webmail.hzau.edu.cn (S.Q.); jiaxianghcao@webmail.hzau.edu.cn (X.J.); zitongGao@webmail.hzau.edu.cn (Z.G.); ZhangWD171215@webmail.hzau.edu.cn (W.Z.); XuQingRong@mail.hzau.edu.cn (Q.X.); 2 Key Laboratory of Preventive Veterinary Medicine in Hubei Province, Wuhan 430070, China; 3 Key Laboratory of Development of Veterinary Diagnostic Products, Ministry of Agriculture of the People’s Republic of China, Wuhan 430070, China

**Keywords:** Porcine deltacoronavirus, Neonatal Fc receptor, polymeric immunoglobulin receptor, NF-κB

## Abstract

Porcine deltacoronavirus (PDCoV) is a porcine enteropathogenic coronavirus that causes watery diarrhea, vomiting, and frequently death in piglets, causing serious economic losses to the pig industry. The strain CHN-JS-2017 was isolated and identified by cytopathology, immunofluorescence assays, transmission electron microscopy, and sequence analysis. A nucleotide sequence alignment showed that the whole genome of CHN-JS-2017 is 97.4%–99.6% identical to other PDCoV strains. The pathogenicity of the CHN-JS-2017 strain was investigated in orally inoculated five-day-old piglets; the piglets developed acute, watery diarrhea, but all recovered and survived. CHN-JS-2017 infection-induced microscopic lesions were observed, and viral antigens were detected mainly by immunohistochemical staining in the small intestine. The neonatal Fc receptor (FcRn) and polymeric immunoglobulin receptor (pIgR) are crucial immunoglobulin (Ig) receptors for the transcytosis ofimmunoglobulin G (IgG), IgA, or IgM. Importantly, CHN-JS-2017 infected five-day-old piglets could significantly down-regulate the expression of FcRn, pIgR, and nuclear factor-kappa B (NF-κB)in the intestinal mucosa. Note that the level of FcRn mRNA in the intestinal mucosa of normal piglets is positively correlated with pIgR and NF-κB. At the same time, the expressions of FcRn, pIgR, and NF-κB mRNA are also positively correlated in infected piglets. These results may help explain the immunological and pathological changes associated with porcine deltacorononirus infection.

## 1. Introduction

Porcine deltacoronavirus (PDCoV) is an enveloped, single-stranded positive-sense (+ssRNA) virus belonging to the family Coronaviridae. PDCoV was first identified in Hong Kong, China in 2012, and subsequently detected on swine farms in the United States, Canada, South Korea, mainland China, Thailand, Laos, and Vietnam; molecular monitoring studies have shown that PDCoV was a common viral pathogen of pigs worldwide [1,2,3,4,5].

The clinical symptoms of PDCoV-infected piglets are like that of other porcine enteric pathogens, such as porcine epidemic diarrhea virus (PEDV) or transmissible gastroenteritis virus (TGEV), including diarrhea, vomiting, dehydration, and mortality [5]. TGEV and PEDV replicate in enterocytes of the small intestine. All PDCoV-infected pigs had PEDV-like lesions characterized by thin and transparent intestinal walls and an accumulation of large amounts of yellow fluid in the intestinal lumen [6]. Further, histology analysis exhibited acute diffuse, severe atrophic enteritis, and mild vacuolation of superficial epithelial cells in the cecum and colon [6]. The PDCoV strain OH-FD22 causes thinning of the intestinal wall, resulting in yellow fluid accumulation in the intestinal cavity [7]. Subsequently, several studies have reproduced similar clinically diarrheal symptoms in gnotobiotic and conventional pigs with isolated PDCoV strains [8,9,10,11]. PDCoV is enteropathogenic in pigs, and lesions are similar but relatively milder than PEDV [12].

The secretory IgA plays a major role in the mucosal anti-infection immunity and is transported by the polymeric immunoglobulin receptor (pIgR). However, recent studies have found that IgG also plays an important role in pathogen infection, which is transported by neonatal Fc receptor (FcRn) [13]. PDCoV is an intestinal pathogen, but little information exists about the regulation of host immune responses due to PDCoV infection. PDCoV infection suppresses interferon (IFN)-β production by inhibiting the retinoic acid-inducible gene I (RIG-I) signaling pathway in Lilly Laboratories Cell–Porcine Kidney (LLC-PK) cells [14]. PDCoV nonstructural protein 5 (nsp5) inhibits IFN-β production by the cleavage of nuclear factor-kappa B (NF-κB) Essential Modulator (NEMO) and cleavage of signal transducer and activator of transcription 2 genes (STAT2) in porcine kidney epithelial (PK-15) cells. In addition, PDCoV N and the accessory protein NS6 were found to antagonize the production of IFN-β in Human Embryonic Kidney (HEK)293T cells [15,16,17]. Although research into these cells has provided invaluable information about the interaction between enteric coronaviruses and their hosts, the research may not yield relevant biological information consistent with in vivo studies. PDCoV induced toll-like receptor TLR3, IL-12, IFN-α, IFN-β, and protein kinase R (PKR) messenger ribonucleic acid (mRNA) expression in infected Peyer’s patches from weaned piglets [18]. However, little is known about the innate immune response to PDCoV infection in vivo. 

In this study, we successfully isolated PDCoV strain CHN-JS-2017 and performed a histological examination and an immunohistochemistry staining analysis to study its pathogenicity in five-day-old piglets. In addition, to better understand in vivo immunological changes after PDCoV infection, we evaluated the effects of PDCoV on the pig intestinal gut pIgR, FcRn, and NF-κB and their correlation by RT-qPCR, which may be related to the pathogenesis of PDCoV.

## 2. Materials and Methods 

### 2.1. Clinical Samples and Virus Isolation

Contents from the intestines of seven PDCoV-positive piglets were collected from a commercial pig farm in Jiangsu Province and analyzed by N-gene based RT-qPCR [4]. These samples were, respectively, homogenized in 10 mL of Dulbecco’s Modified Eagle Medium (DMEM) (Gibco, Grand Island, NY, USA), and the supernatant was collected by centrifugation (4000 *g* at 4 °C for 10 min). The separated supernatant was filtered through a 0.22-μm filter (Millipore, MA) and stored at −80 °C for PDCoV isolation.

Cells were obtained from the China Center for Type Culture Collection (Wuhan, China). LLC-PK cells were cultured in DMEM (Hyclone, USA) containing 10% fetal bovine serum (FBS) (Gibco) and the maintenance medium for PDCoV propagation was DMEM supplemented with 6 μg/mL trypsin (Gibco, USA) in a 5% CO_2_ incubator. LLC-PK cells were cultured in six-well plates to 90% of the cell monolayer, washed three times, and maintained with 0.5 mL filtered inoculum in 1.5 mL of maintenance medium for 2 h. After virus adsorption, the cells were then washed three times with PBS and cultured continuously in 2 mL maintenance medium at 37 °C in 5% CO_2_. At 24 hpi, an obvious cytopathic effect (CPE) was observed in the treated cells; the infected cells were lysed using a freeze-thaw method, and centrifuged (4000 *g* at 4 °C for 10 min). The supernatants were stored at −80 °C. The virus was serially propagated for three passages and titrated in LLC-PK cells. The isolate (designated as CHN-JS-2017) was purified by plaque purification and tested with the median tissue culture infectious dose (TCID_50_) assay protocol, as described previously [7].

### 2.2. Immunofluorescence assay (IFA) 

Immunofluorescence assays (IFA) were performed, as described previously, to observe PDCoV-infected LLC-PK cells [9]. LLC-PK cells infected with PDCoV at a multiplicity of infection (MOI) of 0.01 at 24 hpi were fixed with 4% paraformaldehyde in 24-well plates. Subsequently, PDCoV N-protein polyclonal antibody (1:100) (prepared in our laboratory) was used as the primary antibody, followed by fluorescent isothiocyanate (FITC)-labeled goat anti-rabbit secondary antibody (1:500) (Abclona, WuhanChina), and was then counterstained at room temperature with 4′,6-diamidino-2-phenylindole (DAPI). Fluorescence was examined using a fluorescence microscope (Olympus IX73, Tokyo, Japan).

### 2.3. Western Blot

LLC-PK cells were infected with PDCoV (MOI 0.01) in 24 well-plates at 24 hpi; the cell lysates were prepared for 12% sodium dodecyl sulfate-polyacrylamide gel electrophoresis (SDS-PAGE), and proteins were electroblotted onto a polyvinylidene difluoride membrane (Bio-Rad, USA). FcRn polyclonal antibodies and PDCoV-N polyclonal antibodies were prepared in our laboratory. Mouse mAbs against glyceraldehyde 3-phosphate dehydrogenase (GAPDH) and rabbit polyclonal against phospho-NF-κB and pIgR were purchased from Abclona (China). PDCoV-N, phospho-NF-κB, FcRn, and the pIgR antibody (1:2000) were used as primary antibodies, followed by horseradish peroxidase (HRP)-conjugated goat anti-rabbit immunoglobulin-G (IgG) or anti-mouse IgG (ABclonal, China) as secondary antibodies (1:5000). Proteins were visualized with enhanced chemiluminescence (ECL), as described previously [19]. 

### 2.4. Electron Microscopy

The purified PDCoV-infected LLC-PK cells in six well-plates, harvested at 24 hpi, were negatively stained. The virions were stained with 2% phosphotungstic acid (pH 6.8) for 1.5 min and examined using a Hitachi Model H-7650 transmission electron microscope (TEM). The virus samples were observed according to the methods described in earlier studies [20]. 

### 2.5. Phylogenetic Analysis

Viral RNA was extracted from the isolated third-passage PDCoV strain CHN-JS-2017 with TRIzol™ reagent (Thermo Fisher Scientific, Waltham, MA, USA), and complementary deoxyribonucleic acid (cDNA) was synthesized by using a reverse transcription-polymerase chain reaction (RT-PCR) kit (TaKaRa, Dalian, China). The primers used for the amplification of the genomic fragments of CHN-JS-2017 were described previously (Table S1) [21]. The 5′ and 3′ termini of the genomic sequence were synthesized using rapid amplification of the cDNA ends (RACE) (Vazyme, Nanjing, China). The PCR products were cloned into pMD18-T (TaKaRa, Dalian, China), and sequenced by Sanger sequencing. The genomic fragments were assembled using Lasergene 7.0 (DNASTAR, Inc., Madison, Wisconsin USA) and the assembled genomic sequences were submitted to the GenBank database under accession number MN249445. A phylogenetic tree was performed for the whole genome of the 26 PDCoV strains from different countries using the contiguous method with MEGA 7 software [22].

### 2.6. Inoculation of Piglets with Porcine Deltacoronavirus (PDCoV) Strain CHN-JS-2017

The animal study was approved under the guidance of the Scientific Ethics Committee of Huazhong Agricultural University (HZAUSW-2018-011) and performed in accordance with the committee’s regulations and guidelines. The piglets were randomly divided into two groups (six piglets per group) in separate rooms. The piglets were fed milk powder. After one day of adaptation, the challenged group of piglets were orally inoculated with PDCoV CHN-JS-2017 (5 × 10^6^ TCID_50_/mL, 3 mL/head), while the control group of piglets received 3 mL of maintenance medium orally. Every piglet in both groups was observed daily for lethargy, vomiting, and diarrhea. Fecal swabs for viral RNA detection were collected at 0, 1, 2, 3, 4, 7, 14, 18, and 21 dpi from all piglets. 

Three of six piglets in each group were randomly selected for necropsy at four days post-inoculation (dpi). During the necropsies, fresh jejuna were collected and fixed with 10% formalin for 36 h, and then dehydrated, embedded, sectioned, and mounted onto glass slides. After they were stained with hematoxylin and eosin (H&E), the slides were examined and analyzed with conventional microscopy. The fixed tissue sections were evaluated for PDCoV antigen by immunohistochemistry (IHC) using a PDCoV N-protein polyclonal antibody (1:100 dilution), produced in our laboratory. The immunohistochemistry slides were evaluated by a veterinary pathologist according to the evaluation system of histology and immunohistochemistry, as described previously [23]. RT-qPCR was used to detect the expression of FcRn, pIgR, and NF-κB genes in the porcine duodenum proximal or distal, jejunum proximal or distal, ileum proximal or distal.

### 2.7. Reverse Transcription-Polymerase Chain Reaction (RT-PCR) Analysis

Fecal swabs and small intestine tissues from every piglet were homogenized respectively and centrifuged at 6000 *g* for 5 min. Total RNA was extracted from the supernatant with TRIzol^@^ reagent (Thermo Fisher Scientific, USA); cDNA was synthesized from the extracted RNA by reverse transcription using an RT-PCR kit (Takara, Dalian), and real-time RT-qPCR was performed using the SYBR Green Real-Time PCR Mix (Takara, Dalian). The N gene amplified from the PDCoV CHN-JS-2017 strain was cloned into the pMD18-T cloning vector (Takara, Dalian). The pMD18-N plasmid was serially diluted 10-fold to generate a standard curve in each plate. The viral RNA in the sample was calculated based on the results for the standard curve. Virus titers were detected by Tissue culture infective dose (TCID_50_), as previously described [8]. The specific pig pIgR, NF-κB, FcRn, and GAPDH gene primers were synthesized by GenScript Corporation (Nanjing, China) as described previously (Table S2) [19,24]. The expression level of the gene was calculated relative to the expression of glyceraldehyde 3-phosphate dehydrogenase (GAPDH) using the delta-delta cycle to threshold (2^−ΔΔCT^) method.

### 2.8. Statistical Analysis 

Data were analyzed as mean ± SEM. Differences among groups were performed by one-way ANOVA using Prism (GraphPad Software Inc., San Diego, CA, USA). Pearson’s test was used to assess the correlations between the mRNA levels of different genes in the results. The significance level for all analyses was set as *p* < 0.05 (∗) and *p* < 0.01 (∗∗).

## 3. Results

### 3.1. Virus Isolation and Characterization

**Figure 1:**
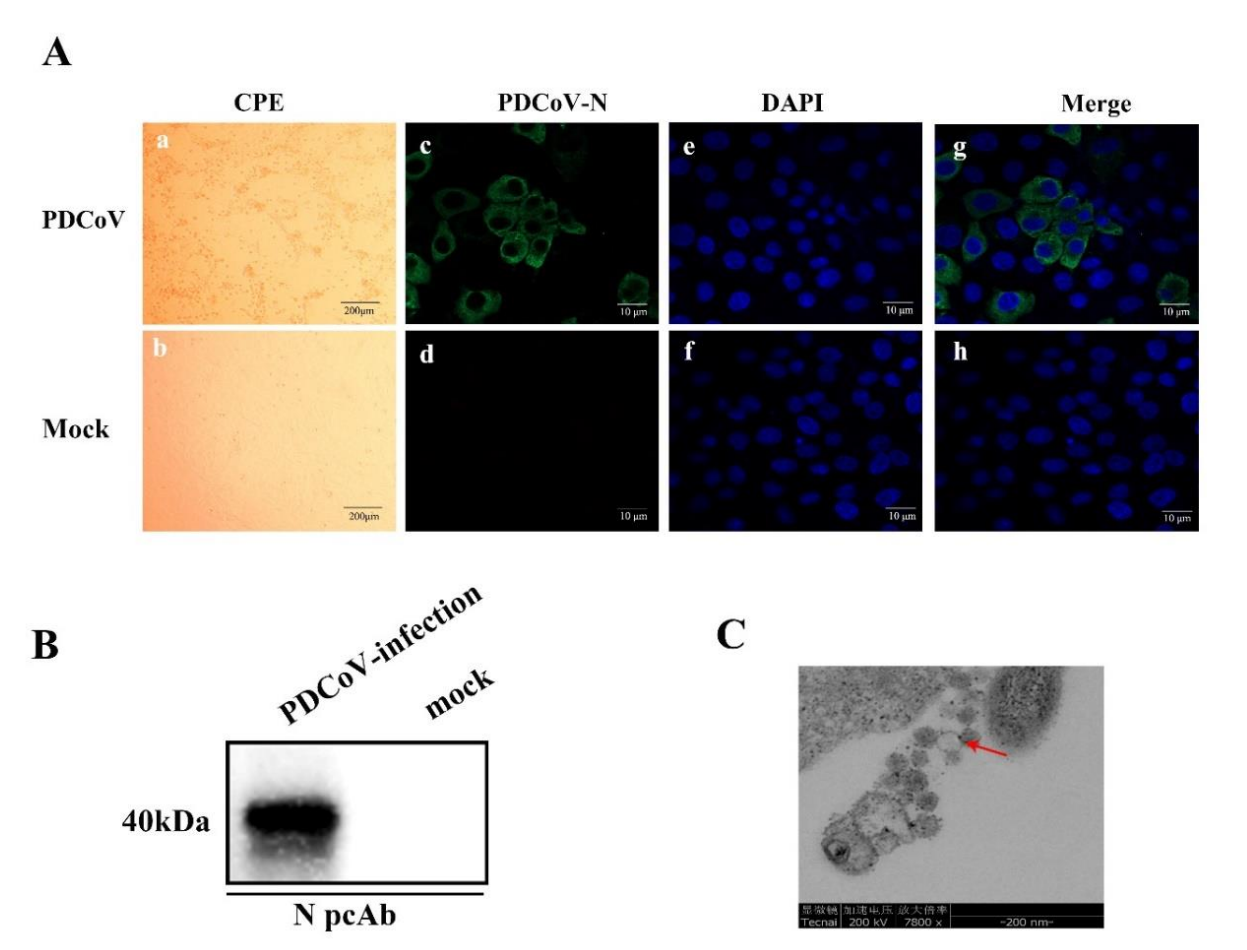
Isolation and characterization of porcine deltacoronavirus (PDCoV) strain CHN-JS-2017. (**A**) The cytopathic effect (CPE) and immunofluorescence staining (IFA) of LLC-PK cells infected with PDCoV. (**a**) CPE of LLC-PK cells at 24 h after PDCoV infection. (**b**) Morphology of the mock-treated LLC-PK cells at 24 h. (**c**–**h**) PDCoV-infected or mock-treated LLC-PK cells were examined by IFA at 24 hpi using polyclonal against PDCoV N protein. (**c** and **d**) IFA with PDCoV N protein (green), (**e** and **f**) IFA with 4′,6-diamidino-2-phenylindole (DAPI)(blue), all views were visualized by using a fluorescence microscope. (**B**) PDCoV-infected or mock-treated LLC-PK cells were subjected to analysis with polyclonal antibodies against PDCoV N protein by Western blot at 24 hpi. (**C**) Electron microscopic image of PDCoV particles. The red arrow points to the virus particle.

Seven PDCoV-positive samples were inoculated onto LLC-PK cells; only one of the isolates showed visible CPE (enlarged, rounded, and aggregated) at 36 h post-infection and was identified as PDCoV CHN-JS-2017(Figure 1A). The PDCoV isolate was confirmed by IFA and Western blot with a PDCoV N-protein polyclonal antibody in the LLC-PK cells. PDCoV N-protein-specific immunofluorescence was detected (Figure 1A), but not in the uninfected group. Western blot analysis also detected N protein in PDCoV-infected LLC-PK cells (Figure 1B). The PDCoV CHN-JS-2017 infected LLC-PK cells were examined by transmission electron microscopy (TEM). Typical crown particles with spiky surface protrusions were observed with negative staining on TEM (Figure 1C). The plaque-purified virus was titrated to 10^6.25^ TCID_50_/mL.

### 3.2. Characterization of the PDCoV CHN-JS-2017 Genomic Sequence

**Figure 2:**
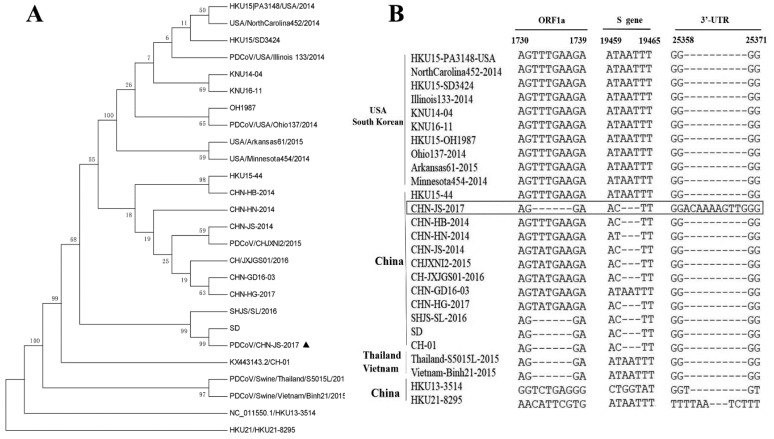
A phylogenetic tree constructed on the basis of the complete genome (**A**) of the PDCoV strain from different countries and local areas. CHN-JS-2017 is represented by a triangle. The phylogenetic tree was constructed by the adjacency method in MEGA 7 (http://www.megasoftware.net). Bootstrap analysis was performed using 1000 replicates. (**B**) Three main deletions or insertions in the complete genome alignment. A multiple sequence alignment was constructed with ClustalW in the DNAStar software. The PDCoV strain CHN-JS-2017 is indicated in bold and highlighted with a box.

A nucleotide sequence alignment showed that the whole genome of CHN-JS-2017(GenBank no: MN249445) is 97.4%–99.6% identical to other PDCoV strains. Phylogenetic analysis indicated that the PDCoV strains from the United States and South Korea clustered into a large clade, whereas CHN-JS-2017 clustered with other PDCoV strains detected in China since 2014 (Figure 2A). These data imply that the Chinese strains might share a more recent common ancestor with the US and South Korean strains than the ancestor of the strains from Thailand and Vietnam. A sequence analysis showed a 6-nt (TTTGAA) deletion at positions 1732 to 1737 in nsp2 (corresponding to the HKU15-44 sequence), 3-nt (AAT) deletion at positions 19461 to 19463 in the S gene, and 10-nt (ACAAAAGTTG) insertion at positions 25360 to 25369 in the 3′-UTR (Figure 2B).

### 3.3. Clinical Observations and Fecal Shedding in Piglets Challenged with CHN-JS-2017

**Figure 3:**
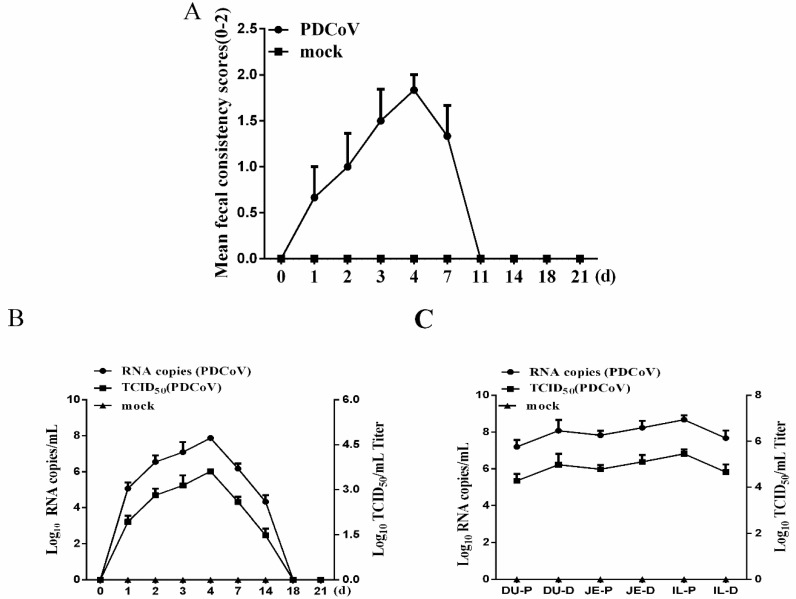
Fecal viral shedding and virus distribution in piglets challenged with PDCoV-JS-2017. (**A**) Diarrhea of piglets in different groups. The severity of diarrhea was scored based on visual examination; 0 = normal and no diarrhea; 1 = mild and fluidic diarrhea; 2 = severe watery diarrhea; scores of 1 or more were considered diarrheic. (**B**) Fecal virus shedding in PDCoV-challenged piglets. (**C**) Virus distribution in the small intestine of piglets challenged with PDCoV. DU-P: Duodenum proximal. DU-D: Duodenum distal. JE-P: jejunum proximal. JE-D: jejunum distal. IL-P: ileum proximal. IL-D: ileum distal.

To assess the pathogenicity of the PDCoV strain CHN-JS-2017 to piglets, five-day-old piglets were challenged with PDCoV CHN-JS-2017. The piglets in the control group were all in a healthy state. Two of the six piglets in the challenge group developed diarrhea at 1 dpi, and the remaining piglets showed lethargy, anorexia, and watery diarrhea at 2–6 dpi, and showed acute diarrhea at 3–6 dpi (Table 1). Severe watery diarrhea gradually recovered afterward. Clinical symptoms were scored according to their level of diarrhea; severe diarrhea was observed in piglets at 3–6 dpi (Figure 3A). Despite watery diarrhea with vomiting, lethargy, and anorexia, no deaths occurred during the study. These results showed that PDCoV CHN-JS-2017 was pathogenic to newborn piglets.

PDCoV RNA was detected by RT-qPCR in feces collected from piglets in the oral challenge group, and all piglets were found to exhibit peak virus shedding of 10^5^–10^8^ copies/mL or 10^4^–10^6^ TCID_50_/mL in feces at 2–6 dpi and high levels in the feces. Virus shedding occurred for about a week; after that, the virus shedding was gradually reduced, reaching a level which was undetectable by RT-PCR at 18 dpi (Figure 3B). The distribution of CHN-JS-2017 in various tissues was also examined by quantitative real-time RT-qPCR in three pigs from each group sacrificed at 4 dpi. Viral RNA was detected in duodenums (average 10^7.64^ copies/mL or 10^5.79^ TCID_50_/mL), jejunum (average 10^8.03^ copies/mL or 10^6.18^ TCID_50_/mL), ileum (average 10^8.16^ copies/mL or 10^6.32^ TCID_50_/mL) (Figure 3C). No PDCoV RNA was detected in the negative control piglets.

### 3.4. Gross Pathology and Histopathology

**Figure 4:**
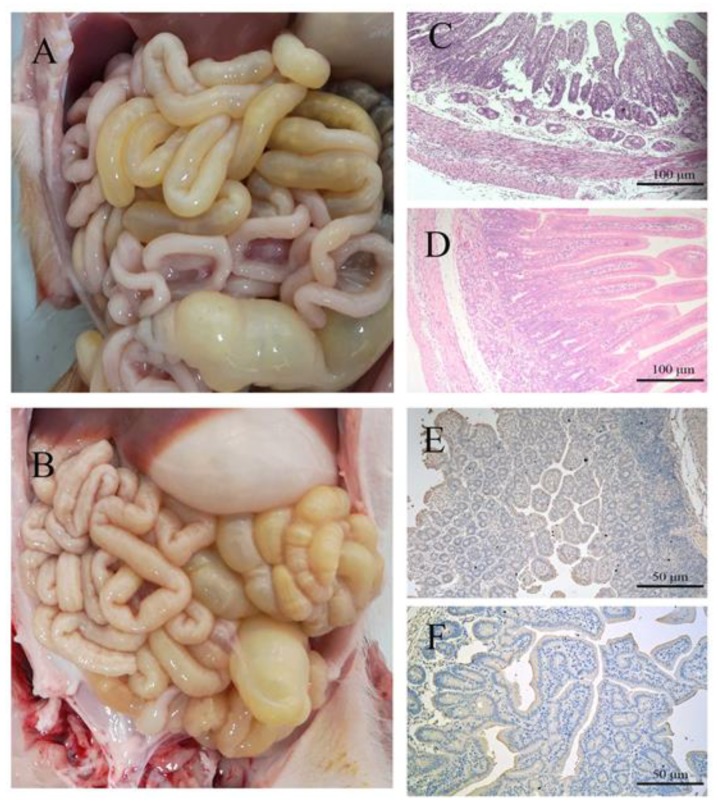
Intestinal changes of infected and uninfected piglets challenged with PDCoV CHN-JS-2017. (**A**) Macroscopic damage of piglets challenged with PDCoV at 4 dpi. (**B**) Macroscopic view of 4 dpi negative control piglets. (**C**,**D**) Hematoxylin and eosin (H&E)-stained jejunal tissue sections of piglets challenged with PDCoV (**C**) or negative control piglets (**D**). (**E**,**F**) Immunohistochemically stained jejunal tissue sections of challenged piglets (**E**) or negative control piglets (**F**).

The overall pathological and histological changes for piglets infected with the PDCoV CHN-JS-2017 strain were determined, and a necropsy performed at 4 dpi. The small intestine (accumulation of large amounts of yellow fluid) was transparent, thin-walled, and gas-swelled (Figure 4A). The control group was normal, and no lesions were observed (Figure 4B). Histopathological examination showed necrosis of small intestinal epithelial cells, atrophy of intestinal villi, and vacuolation in infected pigs compared with the control group (Figure 4C,D). Consistent with the histopathological results, the PDCoV antigen was detected by immunohistochemical staining in the cytoplasm of the infected villous enterocytes compared with the control group (Figure 4E,F). Taken together, these results indicate that PDCoV CHN-JS-2017 may cause intestinal lesions in piglets.

### 3.5. Alteration of NF-κB FcRn and pIgR mRNA in the Small Intestinal Mucosa after PDCoV Infection

**Figure 5:**
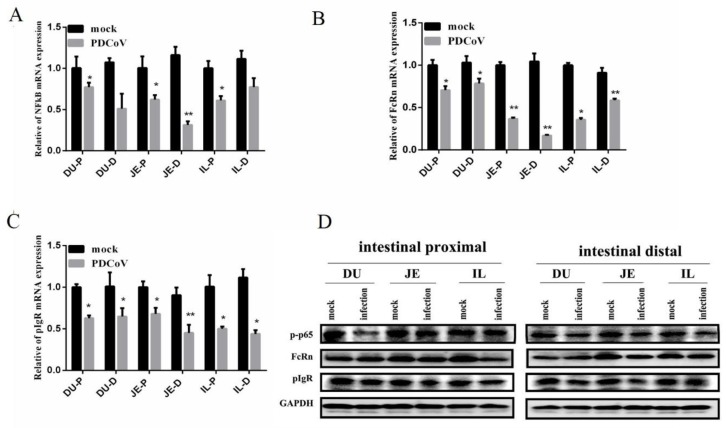
Expressions of nuclear factor-kappa B(NF-kB), neonatal Fc receptor (FcRn) and polymeric immunoglobulin receptor (pIgR) mRNA expression in the small intestinal mucosa of piglets induced by PDCoV CHN-JS-2017 at 4 dpi. The mRNA levels of NF-kB (**A**), FcRn (**B**), and pIgR (**C**) in the small intestinal mucosa were determined individually in each animal by RT-qPCR. All data are expressed as the mean ± SEM of three independent experiments. * *p* < 0.05, ** *p* < 0.01, relative to the mock-treated piglets. Relative amplification of the target genes expression levels was normalized to GAPDH expression. (**D**) The protein levels of NF-kB, FcRn, and pIgR at the small intestinal mucosa were determined individually in each animal by Western blot. DU-P: Duodenum proximal. DU-D: Duodenum distal. JE-P: jejunum proximal. JE-D: jejunum distal. IL-P: ileum proximal. IL-D: ileum distal.

To characterize the effects of PDCoV infection, the levels of NF-κB FcRn, and pIgR in the intestinal mucosa of infected or non-infected control piglets were examined. The mRNA levels of NF-κB, FcRn, and pIgR mRNA in the intestinal mucosa of piglets were statistically significantly reduced (*p* < 0.01) compared to the control group, especially in the jejunum (Figure 5A–C). Moreover, the levels of NF-κB, FcRn, and pIgR in the jejunum proximal (JE-P) levels of mRNA were significantly lower than in the jejunum distal (JE-D). Similar, the protein expression of NF-κB, FcRn, and pIgR were found to be consistent with the results of RT-PCR (Figure 5D).

### 3.6. Correlation between NF-κB and FcRn or pIgR in the Small Intestine Mucosa in Infected or Non-Infected Control Piglets

As shown above, the mRNA levels of FcRn, pIgR, and NF-κB genes tend to be lower after PDCoV infection. It has been reported that NF-κB is involved in the regulation of FcRn and pIgR expression in mice. Therefore, the correlation between the mRNA levels of these genes was examined. 

**Figure 6:**
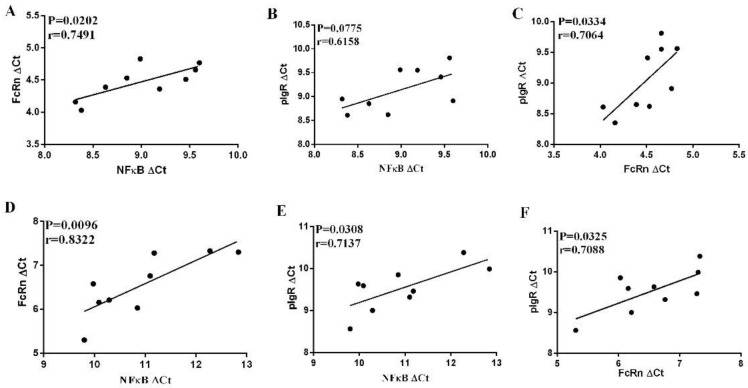
Correlations between the mRNA levels of NF-kB, pIgR and FcRn in the intestinal mucosa of infected piglets. Significant positive correlations were found between NF-kB and FcRn (**A**) and FcRn and pIgR (**B**), but not between NF-kB and pIgR (**C**) in uninfected piglets; however, significant positive correlations were found between NF-kB and FcRn (**D**), FcRn and pIgR (**E**), and NF-kB and pIgR (**F**) in infected piglets. FcRn ΔCt = Ct _FcRn_ − Ct_GAPDH_, NF-kB ΔCt = Ct _NF-kB_ – Ct _GAPDH_, pIgR ΔCt = Ct _pIgR_ – Ct _GAPDH_.

The levels of NF-κB mRNA were positively correlated with FcRn mRNA levels in uninfected piglets (*p* < 0.05) (Figure 6A), while no significant correlation was observed between NF-κB and pIgR mRNA levels in uninfected piglets (*p* > 0.05) (Figure 6B). In addition, the mRNA levels of FcRn were positively correlated with those of pIgR in uninfected piglets (*p* < 0.05) (Figure 6C). Notably, the levels of NF-κB and pIgR or FcRn mRNA were positively correlated with each other in infected piglets (*p* < 0.05) (Figure 6D,E). The levels of FcRn mRNA were positively correlated with pIgR mRNA in the small intestine mucosa of infected piglets (Figure 6F). 

## 4. Discussion

Since the first detection of PDCoV in piglet diarrhea in the United States in 2014—the first confirmed pathogenicity in pigs [4,23]—this new type of porcine intestinal coronavirus has attracted attention worldwide. In mainland China, the prevalence of PDCoV in pigs is 33.71% in Jiangxi Province [25], 23.4% in Guangdong Province [26], and 10.2% in Hebei Province [27]. Although the prevalence of PDCoV worldwide is confirmed, the isolation rate of PDCoV is low. In this study, we successfully isolated the CHN-JS-2017 strain of PDCoV (which is highly pathogenic to piglets) and conducted the preliminary exploration of its pathogenic characteristics.

The isolation of PDCoV is difficult, and the addition of trypsin is essential for the isolation of the virus. We attempted to isolate the virus from the intestinal contents of seven PDCoV-positive pigs using LLC-PK cells. Obvious lesions were observed 24 hours after inoculation in the challenged group and confirmed by IFA and TEM. Only PDCoV CHN-JS-2017 was successfully isolated. Many researchers have tried to isolate PDCoV, but the relatively low success rate may be related to the type of disease, the freshness of the sample, the amount of trypsin added to the cells, and the preservation conditions of the sample.

To characterize viral isolates, genome-wide sequencing and multi-sequence alignment and phylogenetic analysis are needed. All known PDCoV strains have high levels of nucleotide sequence identity. It is worth noting that the PDCoV CHN-JS-2017 strain can be clustered into a clade with other strains from mainland China, indicating that the current PDCoV strains are prevalent in many provinces in China. The PDCoV strain CHN-JS-2017 genome is more like the strains from China, the United States, and South Korea than the Thailand strain, while the PDCoV strains from Thailand and Vietnam are clustered in another clade. However, whether these unique variants contribute to the efficiency and virulence of viral replication requires further investigation. Pending further validation, mutations in the nsp2 and S gene imply that these unique variations contribute to the enhanced virulence of this strain. Additionally, a 10-nt insertion in this strain was found in the 3′UTR, which is different to the other PDCoV strain; the PDCoV strain HKU21-8295 from the common-moorhen also contained this 7-nt insertion, and HKU13-3514 from munia also contained this 1-nt insertion, indicating that these insertions in the 3′UTR may be related to cross-species transmissibility.

It is worth noting that several groups have confirmed the pathogenicity of conventional pig PDCoV strains [8,9,10,11]. Overall, these results confirm that PDCoV causes severe diarrhea and vomiting in pigs aged 5–21 days. We used the PDCoV CHN-JS-2017 strain to infect five-day-old newborn piglets by oral feeding. Our success in inducing severe diarrhea and vomiting in piglets through oral infections strongly suggests that PDCoV poses a significant threat to newborn piglets on pig farms. In addition, we collected fecal swabs from piglets challenged with PDCoV and detected viral fecal shedding with real-time PCR. PDCoV RNA was detected from 1–14 dpi, and no RNA were detected in the control group piglets during the study period. This indicates that PDCoV infection by fecal-oral contamination may be the main route of transmission for piglets. In addition, during the necropsy on the seventh day, large lesions were clearly observed in the small intestine of the piglets [9]. In an earlier report, microscopic lesions were observed in all parts of the small intestine infected with PEDV [28]. In the current study, we observed microscopic lesions of the jejuna of CHN-JS-2017 infected piglets, indicative of PDCoV. The disease caused by PDCoV is milder than the disease caused by PEDV. In addition, the PDCoV antigen in the cytoplasm of the villous intestinal cells of the piglets challenged with PDCoV was detected by immunohistochemical analysis. Taken together, these results confirm that the PDCoV CHN-JS-2017 strain isolated in this study may cause intestinal disease in newborn piglets.

IgA and IgG play a major role in mucosal infection immunity; some cytokines or pathogens have also evolved ways to regulate pIgR and FcRn to facilitate infection [29,30]. Reovirus, LPS (a ligand for Toll-like receptor 4 (TLR4) TLR4), IFN-γ, and tumor necrosis factor-alpha (TNF-α) all regulate pIgR and FcRn expression, mainly by the activation of the NF-κB or Janus kinase/signal transducers and activators of transcription (JAK-STAT) JAK-STAT pathway [31,32,33,34]. The down-regulation of FcRn and pIgR by reovirus have been shown in the tracheal mucosa of simian-human immunodeficiency virus/simian immunodeficiency virus (SHIV/SIV)-infected rhesus monkeys [35,36]. Human cytomegalovirus evades humoral immunity by the degradation of FcRn [30]. TGEV-induced FcRn expression by activating NF-κB signaling in porcine small intestinal epithelial (IPEC-J2) cells [19]. However, PDCoV CHN-JS-2017 significantly down-regulated FcRn, pIgR, and NF-κB expressions, and they were positively related to each other in infected piglets. Therefore, the reduced levels of FcRn and pIgR mRNA might be attributed to the reduced levels of NF-κB. In addition, PDCoV might reduce the ability of FcRn and pIgR to transport PDCoV-specific IgG and IgA across the mucosal epithelium, resulting in impaired intestinal immunity in the mucosa, reducing the host’s anti-infection ability. These data demonstrated the altered expression of FcRn and pIgR in coronavirus, and by extension, coronavirus infections, which might have contributed to providing another insight into the immune escape strategy of PDCoV. 

In summary, we successfully isolated and characterized PDCoV CHN-JS-2017 in cultured cells, and CHN-JS-2017 is pathogenic in piglets. Furthermore, the inoculated piglets showed down-regulated FcRn and pIgR expression by NF-κB mRNA. At the same time, we found that the expression of FcRn and pIgR of piglets infected with PDCoV is positively correlated with NF-κB. The results help us to understand the molecular epidemiology and pathogenesis of PDCoV and also provide insights into the development of effective vaccines against this significant pathogen.

## Figures and Tables

**Table 1:** Clinical observation records of five-day-old pigs challenged with PDCoV.

dpi(d)	Lethargy, Vomiting and Anorexia	Fecal Consistency		
		Normal	Mild Diarrhea	Watery Diarrhea
**0**	0/6	6/6	0/6	0/6
1	2/6	4/6	1/6	1/6
2	3/6	3/6	1/6	2/6
3	4/6	2/6	1/6	3/6
4*	3/3	0/3	2/3	1/3
7	2/3	1/3	1/3	1/3
8-21	0/3	3/3	0/3	0/3

* Three pigs were necropsied at four days post-inoculation.

## Supplementary Materials

The following are available online at https://www.mdpi.com/1999-4915/12/1/79/s1. Table S1. Primers for amplification of the PDCoV genomic fragments by RT-qPCR. Table S2. Primers used for RT-qPCR analysis of the gene expression of piglet intestinal mucosa.
