# Supplementary material for: Isolation and Identification of Porcine Deltacoronavirus and Alteration of Immunoglobulin Transport Receptors in the Intestinal Mucosa of PDCoV-Infected Piglets
Source: Viruses. 2020 Jan 9;12(1):79. doi: 10.3390/v12010079 (PMC7019308; doi:10.3390/v12010079)
Supplement: Supplementary file 1 [file viruses-12-00079-s001.pdf]

Table S1. Primers for amplifications of the PDCoV genomic fragments by RT-qPCR.

Name	Sequence(5'to3')	Position in PDCoV genome	Size(bp)
1F	GTTCTCTACCGACACCAATCCA	64–85	1800
1R	GGGTAAGATCCTGGAGTTCACG	1842–1863	
2F	ATCTTGGGCTATCATTGAGCGT	1706–1724	1587
2R	CCTACATCAGTGCTGTCCGTT	3271–3292	
3F	AGTACCAGTAACACCTGCGGAT	3074–3095	1802
3R	CTACCCAAAACACTCCACGACT	4854–4875	
4F	TCTGGACATTTTGGATCTTTGGA	4805–4827	1714
4R	GATGCGAGTCAAGGGCTGTGTT	6497–6518	
5F	AGTCAGCAGGCTATACGTGTGA	6399–6420	1785
5R	GCGTGGGCAGTAGACAACATTC	8162–8183	
6F	GGAGGCGGTTCACAGTTGTA	7995–8014	1807
6R	CAGCAATGTTACGGTTTCCACC	9780–9801	
7F	AGTTAAGATGTCCCCACCGC	9713–9732	1878
7R	TTAGGTAAAGGCAGGGCACT	11567–11586	
8F	TAACGGGTTCTAGTGACGCC	11418–11437	1747
8R	TGTTCCAATCACGATGGTCTGA	13144–13164	
9F	TGCCTACACTCACTCAGATGA	12999–13019	1886
9R	CGGTGTAACAACATGTGCCAT	14864–14884	
10F	GCTAAAACAGATGGCAGTGACA	14783–14804	1850
10R	TTAGTGAAACAAGCCCGCCT	16613–16632	
11F	TGCTTTGTCACTTGGGCTCA	16526–16545	1797
11R	GCTTGCGTTAGGTGAGGTAAC	18302–18322	
12F	TTGGCGGAACTCACACACTT	18210–18229	1800
12R	TGACCCCGATACAACCTAACA	19989–20009	
13F	GTGAGCAGTTTAACTACACCACT	19796–19818	1711
13R	TTCTCAGCATCAACAACACCA	21486–21506	
14F	AGCAGCATACTAACCACCAGA	21310–21330	1745
14R	ACTAGGGTGAAGGGTTGGAGCA	23032–23054	
15F	ACCAACCAACACCGTCCTTT	22768–22789	1503
15R	GGGGTATCATTAGGAGGGAGT	24250–24270	
16F	GACCCTCTCGTTAAAACTCGC	23784–23804	1639
16R	GCTCCATCCCCCCTATAAGCCAA	25400–25422	
5′RACE	GCAACACAATACGCCCAGAT	454–473	
3′ RACE	AGCCACCTACCAAACCAACT	24870–24899	

Table S2. Primers used for RT-qPCR analysis of genes expression of piglet intestinal mucosa

gene name	Primer Sequence (5′−3″)	Amplicon Length	Accession number
GAPDH-F	GGAAAGGCCATCACCATCTT	85 bp	XM_021091114.1
GAPDH−R	CATGGTCGTGAAGACACCAG		
FcRn−F	GGCGACGAGCACCACTACTG	88 bp	HQ026019.1
FcRn−R	AGCCGACCATGATTCCAACC		
pIgR-F	AGCCAACCTCACCAACTTCC	105bp	AB032195.2
pIgR-R	CTGCTAATGCCCAGACCAC		
NF-κB(p65)-F	ACATGGACTTCTCAGCCCTTCTGA	285 bp	CN155798.1
NF-κB(p65)-R	CCGAAGACATCACCCAAAGATGCT		
